# Improving Stability and Accessibility of Quercetin in Olive Oil-in-Soy Protein Isolate/Pectin Stabilized O/W Emulsion

**DOI:** 10.3390/foods9020123

**Published:** 2020-01-23

**Authors:** Qiang Wang, Huaheng Wei, Chaofang Deng, Chenjing Xie, Meigui Huang, Fuping Zheng

**Affiliations:** 1 Beijing Advanced Innovation Center for Food Nutrition and Human Health, Beijing Technology and Business University, Beijing 100048, China; wangqiang@cque.edu.cn; 2 Cooperative Innovation Center of Lipid Resources and Children’s Daily Chemicals, Chongqing University of Education, Chongqing 400067, China; wh1789034295@163.com (H.W.);; 3 College of Light Industry and Food Science, Nanjing Forestry University, Nanjing 210037, China; Cjxie1994@163.com (C.X.); huangmgnj@hotmail.com (M.H.); 4 Beijing Laboratory of Food Quality and Safety, Beijing Technology and Business University, Beijing 100048, China

**Keywords:** quercetin, soy protein isolate, olive oil, Pickering emulsion, pH impacts

## Abstract

Herein we report a soy protein isolate/pectin binary complex particle to stabilize emulsion (olive oil served as dispersed phase) containing quercetin. FTIR was conducted to confirm successful preparation of emulsion before and after embedding quercetin. CLSM was used to determine the microstructure and zeta-potential, rheological behavior, storage stability and freeze-thaw stability were analyzed and were correlated with pH condition. Olive oil-soy protein isolate/pectin emulsion at pH 3.0 can remain stable after 30 days’ storage and exhibited greatest freeze-thaw stability after 3 cycles. Quercetin availability was evaluated by in vitro gastrointestinal digestion experiments and it reached 15.94% at pH 7.0.

## 1. Introduction

As a major dietary intake flavonoid, quercetin is a multi-hydroxyl analog featuring a good potential for chelating metals, scavenging radicals, impacting on enzyme and gene expression [1]. Thus, quercetin exhibits favorable antioxidative, anticarcinogenic, anti-inflammatory, anti-aggregatory and vasodilating capability that makes it promising application in functional food, cosmetic, pharmaceutical industry [2,3]. Quercetin is widely present in plants, such as onions, black tea, apples and berries and so forth. However, quercetin has poor bioavailability due to its hydrophobicity and high instability when physical and chemical condition changes, therefore the use of quercetin as nutraceutical has been limited [4]. Accordingly, how to improve the solubility and stability of quercetin has attracted much attention. Increasing number of approaches have been reported and efficient delivery systems were designed to increase the solubility, stability and absorption of quercetin. Fabricating polymer nanoparticles, like bovine serum albumin [5], soy protein isolate [6], zein nanoparticles [7], synthesized poly (lactic acid) (PLA) [8], has successfully improved quercetin bioavailability under acidic, neutral, alkaline or even thermal, conditions. Besides, excipient foods [9], inclusion complex [10,11] and micelles [12] also served as delivery method to ameliorate quercetin’s solubility and biological activity.

Solid particle stabilized Pickering emulsions exhibit excellent resistance to creaming and coalescence benefiting from high attachment energy between the particles that are irreversible absorbed at the emulsion interface [13,14]. Pickering emulsions have a good deal of merits, such as requires less emulsifier, with no surfactant, easy to preparation, low cost and environment benign. Remarkably, Pickering emulsions can maintain stable being invulnerable to external conditions. Thus, Pickering emulsions are widely used in pharmaceutical, food additive, cosmetics systems [15]. Often solid particles used to stabilize Pickering emulsions are artificial chemicals which may lead to biotoxicity and the applicability of Pickering emulsions in food industry would therefore be limited. Plant protein is a potential solid particle for application in emulsions as its bioavailability. Based on the long-term exploration of the construction, microstructure characteristics and self-assembly of plant protein colloidal particles, Pickering emulsion stabilized by protein particles have been developed in recent years. Zein [16], whey protein [17,18], soy protein [19] and so forth, were widely investigated. It is believed that protein particles irreversibly absorbed at the oil-water interface can modify and rebuild the surface and therefore stable three-dimensional networks formed to keep the emulsion stable for a long time [20].

Soy protein isolate (SPI) is a commercially available nutriment containing nine essential amino acids, flavonoids, vitamin E, saponins and other functional physiological active compounds that all are beneficial to the human body [21,22]. SPI dominantly consists of globulins, like glycinin (11S) and b-conglycinin (7S), which accounts for 34% and 27% of the total protein content of SPI [23], respectively. Soy protein isolate can be used in food processing to improve the quality and texture of food. Besides, it has been found that soy proteins can decrease the interfacial tension between the water and oil and help stabilize emulsions by forming a physical barrier at the oil/water interface [24] resulting in excellent functional properties such as solubility, emulsification, foaming, gelation and aggregation [25]. However, external conditions such as pH, ionic strength or the presence of other components will impact on its solubility and stabilizing behavior in food emulsions [26]. In particular, under acidic conditions, solubility of SPI will obviously decrease which would limit the usage of SPI as an emulsifier. Despite solubility, variable structure of SPI limits its usages as well [27]. Using electrostatic interaction between protein and polysaccharide to formulate complex particle is a powerful and eco-friendly method to modify the protein for applying as a viable emulsifier.

Pectin is a natural nutrient and a green additive in human food recommend by FAO and WHO with no intake limitation. Composed of 80% galacturonic acid, pectin is an acidic polysaccharide and is one of the most commonly used polysaccharides in acidic environment [28]. Pectin shares physiological activities, like help to reduce blood sugar and cholesterol, to augment spleen cells and stimulate macrophages and also can prevent cancer [29]. Moreover, exhibiting excellent gelling function pectin is often used as gelling agent, thickener and quality improver in food processing [30,31,32]. As gel agent, pectin can help to slow down the movement of droplets, to reduce the collision frequency and coalescence rate by increasing the viscosity or forming gel network in continuous phase, which is conducive to the stability of emulsion. Complexing pectin with soy protein isolate is a promising solution to improve stability of SPI under acidic condition by means of the hydrogen-bond formed between protein and pectin generating a strong gel network.

Emulsification strategy also has a critical role on stability of emulsion. Dispersion and particle size of droplets are essential factors for the stability, appearance, structure, rheological behavior of emulsion. Unlike conventional emulsifying procedure, Kobayashi prepared monodispersed stable emulsion by using microchannel emulsification [33]. More researchers [34,35,36,37] used ultrasonic treatment to obtain small droplet size emulsion by benefiting from ultrasonic cavitation function [38]. It was reported that ultrasonic treatment can improve surface hydrophobicity, surface activity and protein aggregation. Stable interfacial protein films formed by using ultrasonic help promote the stability of emulsifiers.

In this work, we prepared soy protein isolate/pectin complex particle stabilized emulsion to efficiently encapsulating quercetin by ultrasonic at series of pH condition to generate an environmental friendliness, wholesome and stable emulsion. Microstructure, particle size, zeta-potential, rheological behavior, storage stability and freeze-thaw stability were studied as a function of pH value to explore the most stable condition. In vitro digestion was also investigated to evaluate the bioavailability of quercetin encapsulated in this olive oil -SPI/pectin(O/W) emulsions. 

## 2. Materials and Methods 

### 2.1. Materials

Soybean protein isolate, pectin, quercetin and methanol were purchased from Shanghai Yuanye Biotechnology Co., Ltd., Shanghai, China. Extra olive oil was donated by Chongqing Jiangyuan Olive Development Co., Ltd., Chongqing, China. Hydrochloric acid and sodium hydroxide were obtained from National Medicine Group Chemical Reagent Co., Ltd., Shanghai, China. Artificial gastric fluid, artificial intestinal fluid, artificial saliva, Nile red dye and Nile blue dye were provided by Beijing Leigen Biotechnology Co., Ltd., Beijing, China.

### 2.2. Emulsion Preparation

SPI/pectin complex particle dispersions were first prepared by blending SPI aqueous dispersions (5.0% protein *w*/*w*) with specified concentrations of pectin samples (1.0% *w*/*v*) in SPI/pectin ratio of 1/1 (*v*/*v*). SPI/pectin complex particle solutions were then homogenized with olive oil by ultrasonic for 6 min (Ultrasonic cell grinder, SCIENTZ-IID, Ningbo Xinzhi Biotechnology Co., Ltd., Ningbo, China) to generate the resultant O/W emulsions (oil volume fraction 50%). Then, 2.0 mL quercetin (0.1 mg/mL) was dissolved in olive oil and added to the preceding SPI/pectin complex to prepare the ultimate SPI/pectin complex particle stabilized emulsions with quercetin loaded. The pH of the samples was adjusted to pH 3.0, pH 5.0, pH 7.0, pH 7.5, pH 9.0 by using HCl (0.01 M) or NaOH (0.01 M) with a pH meter monitor (BPH-7200, Beier analytical Instruments Co., Ltd., Dalian, China).

### 2.3. Microstructure of Emulsion

The microstructure of the emulsions was first evaluated by optical microscopy (Axio Lab.A1 pol, Carl Zeiss Jena, Jena, Germany) and then by confocal laser scanning microscopy (CLSM, LSM710, Carl Zeiss Jena, Jena, Germany). For optical microscopy observations, the samples were dyed with red ink and for CLSM observations, the samples were dyed with Nile Blue for proteins and Nile Red for oil. 0.1 g/L Nile Blue (or Nile Red) in propylene glycol was added into the emulsions for 40 min in dark to obtain the test samples which were then partly put on concave slides and were observed by using a 40× magnification lens with laser excitation wavelength at 514 nm for Nile Red and 633 nm for Nile Blue. For each sample, three images were captured randomly and droplets (generally 100) in each image were measured to get their dispersion and average size by using Image J method.

### 2.4. Zeta-Potential

The zeta-potential of emulsions was determined using a Zetasizer JS94HM (Micromeritics Instrument Ltd., Norcross, GA, USA). Emulsions were diluted to a protein concentration of approximately 0.5 wt % using deionized water. 

### 2.5. Rheology Analysis

The rheological characterization was calculated using a microscale infrared combined rheometer (MARS60, Thermo Fisher, Karlsruhe, Germany) according to the process followed by previous procedures with some modifications [39]. First, 3 mL of the molten CS-ATCs-KC was poured onto a parallel plate (approximately 25 °C) with a diameter of 30 mm (with a gap of 1mm) to facilitate the formation of the initial gel. Then, a thin layer of silicone oil was applied to the rim of the sample to prevent evaporation. More details about the test condition is provided below. The shear rate was ranging from 0.1 to 100 S^−1^; the stress was ranging from 0.1 to 1000 Pa and the frequency was fixed at 1 Hz; and the frequency was ranging from 0.1 to 10 Hz and the stress was fixed at 1 Pa.

### 2.6. Freeze-Thaw Protocol

Fresh emulsions were charged in tubes to be centrifuged at 10000 rpm for 10 min and then isothermally stored at −20 °C for 24 h. Subsequently, the frozen samples were thawed at room temperature for 3 h. This cycle was repeated three times and the appearance of the samples (distinguished by H_pH3.0_, H_pH5.0_, H_pH7.0_, H_pH7.5_, H_pH9.0_) were investigated at each cycle.

### 2.7. Creaming Index 

Samples after storage process or freeze-thaw cycles in the tube were separated into several layers, the height of each layer was measured by using a ruler. The creaming index (CI) was given by the height of the serum layer (Hs) and the total height of the emulsion (Ht)

CI (%) = HS/HT × 100%.(1)

### 2.8. FT-IR Analysis

Fourier transform infrared (FT-IR) characterization of the particles was conducted according to the method of Navikaite et al. (2016) with minor modifications. All samples were freeze-dried and recorded on a Fourier-transform infrared spectrometer with a single-reflection attenuated total reflectance (ATR) accessory (VERTEX 80V, Bruker, Ettlingen, German)—the measurement range was 500 cm^−1^~4000 cm^−1^, scan times—32, time-resolved spectrum—500 picoseconds, fast scan—8 ms, resolution ratio—0.07 cm^−1^, SNR—40,000:1.

### 2.9. Measurement of Quercetin Encapsulation Efficiency

Ten milligram quercetin was dissolved in 100 mL 80% methanol (*v*/*v*) and then diluted to get a constant-gradient concentration of the quercetin solution. Methanol (80%, *v*/*v*) served as blank sample and then scan the quercetin solution series at a wavelength range of 230~800 nm using an ultraviolet spectrophotometer. The calibration curve was plotted with absorbance (as ordinate) at 373 nm of maximum wavelength and standard mass concentration of quercetin (as abscissa). Ultraviolet spectrophotometer (UV mini-1240 Mettler Toledo Instruments Co., Ltd., Shanghai, China) was used. Immediately after quercetin encapsulated emulsions were prepared, the encapsulation efficiency was determined by extraction method as described in our previous study [40], the products were centrifuged into a refrigerated centrifuge (sigma 2-16k, Boli Instruments Co., Ltd., Beijing, China) at 11,000 rpm for 25 min in order to separate the unencapsulated quercetin. The mass of free quercetin in the tube was analyzed according to a quercetin standard curve, through the measurement of maximum absorbance at 373 nm by ultraviolet spectrophotometer. The quercetin loading efficiency in SPI/pectin complex particles was calculated as follows:

Encapsulation Efficiency (%) = (1 − free quercetin/gross quercetin) × 100%.(2)

### 2.10. In Vitro Gastrointestinal Digestion

First, 4 g pre-incubated quercetin encapsulated emulsions (with different pH, at 37 °C) were mixed with 5.0 mL artificial gastric fluid at pH 2.0 for 2 h. Then, the pH of the mixture was adjusted to 7.0 by using NaOH (0.002 M) and 10 mL artificial intestinal fluid, 20 mL PBS (pH 7.0) buffering solution were added. During the 2-h intestinal digestion, the pH was monitored and always kept at 7.0 by using NaOH to neutralize free fatty acids (FFA) released as a result of lipid digestion. The percentage of free fatty acids released during digestion was calculated by the following equation:

(3) $$Y_{1}\left(\%\right)=\frac{c_{NaOH}\times V_{NaOH}\times M_{lipid}}{2\times m_{lipid}}\times 100\%$$

where $Y_{1}$ is the release rate of free fatty acid, $c_{NaOH}$ is the concentration of NaOH, $V_{NaOH}$ is the volume of NaOH used intended to keep the pH at 7.0, $M_{lipid}$ is the average mass of lipids per unit volume, $m_{lipid}$ is the gross mass of lipids in the emulsion.

After all the stages, the original mixture was centrifuged at 11,000 rpm for 25 min at 4 °C and the aqueous layer containing quercetin was collected for absorbance measurement. And the bioavailability of quercetin is given by the amount of quercetin left after digestion/the original amount of quercetin encapsulated in the emulsions. 

### 2.11. Statistical Analysis

Generally, data were repeated at least three times and evaluated by their means and standard deviations. Data are expressed as mean value ± standard deviation. All data were executed significance test by using IBM SPSS Statistics 22 and Tukey test to determine significant differences between means (*p* < 0.05). 

## 3. Results and Discussion

### 3.1. FT-IR Spectrometer Analysis of SPI/Pectin Complex and Olive oil-SPI/Pectin Emulsion

**Figure 1:**
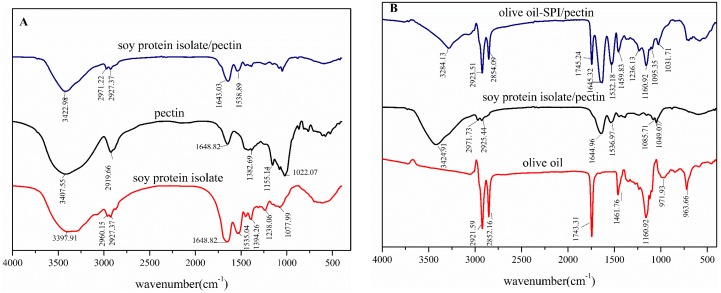
Infrared spectrum of (**A**) soy protein isolate, pectin and its complexes; and (**B**) olive oil-soy protein isolate (SPI)/pectin Pickering emulsion, olive oil, soy protein isolate/pectin complex solution.

FT-IR spectroscopy is a helpful tool to identify compound structure by absorption peak of distinguishable functional groups and to trace structural changes. According to literature [41], absorption bands 1600–1700 cm^−1^, 1450–1550 cm^−1^ and 1200–1450 cm^−1^ refers to amide I band (N-H bending), amide II band (C-N stretching) and amide III band (C-N stretching and N-H bending) respectively. For SPI here, as shown in Figure 1A, absorption at 1649 c^−1^ contributed to C=O stretching in amide I and 1535 cm^−1^ to N-N bending in amide II; 1394 cm^−1^ is consistent with C-N stretching and N-H bending in amide III band. Pectin molecule can also be easily discerned, as Figure 1 presented, peak at 3407 cm^−1^ is formed by O-H stretching, 2919 cm^−1^ by C-H stretching on the sugar ring, 1680–1600 cm^−1^ by asymmetric stretching of C=O in free carboxylic acid and -OH, -CH bending lead to absorption at 1649 cm^−1^, 1383 cm^−1^ respectively. In particular, absorption at 1155 cm^−1^ refers to C-O-C stretching, indicating the presence of the methoxyl group. The FT-IR spectroscopy clearly suggested that the SPI and pectin was successfully conjugated for the disappearance of absorption both at 3398 cm^−1^ from SPI and 2919, 3407 cm^−1^ from pectin and new formed peak at 3423cm^−1^ were observed, indicating that the electrostatic interaction occurred between the amino from protein and the hydroxy from pectin. Similarly, the FT-IR spectroscopy (Figure 1B) tells us successful formulation of olive oil-SPI/pectin Pickering emulsion. In the spectroscopy of emulsion particles, it presented all identifiable absorption peak from those three compounds with slight shift though. We identified all peaks from low absorption band to high band one by one. This is, strong absorption at 1161 cm^−1^ is consistent with C-O-C stretching from pectin, 1460 cm^−1^ from olive oil, 1532 cm^−1^ formed by N-H bending from amide II in soy protein isolate, 1745 cm^−1^ from C=O stretching in olive oil molecule, peak at 2854 cm^−1^ caused by C-H stretching from olive oil alkyl skeleton and 2923 cm^−1^ is concurrently led by C-H stretching both in olive oil and pectin molecule. Figure 1 indicated that SPI/pectin complex particles and olive oil involved Pickering emulsions were prepared successively.

### 3.2. Zeta-Potential of SPI/Pectin Complex and Olive Oil-SPI/Pectin Emulsion.

**Figure 2:**
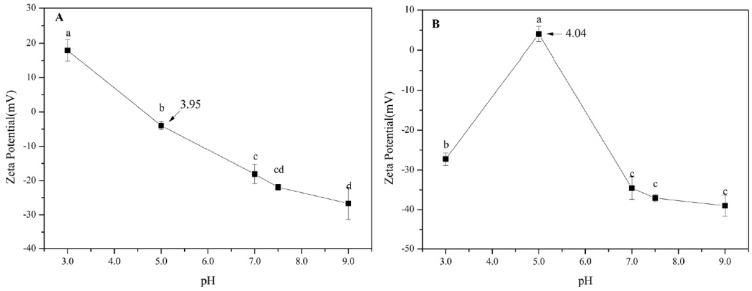
Zeta-Potential of (**A**) SPI/pectin solution and (**B**) olive oil-SPI/pectin Pickering emulsion at different pH values. Data represent the mean ± one standard deviation (*n* = 3). Different lower-case letters represent a significant difference among pHs.

Stability of emulsions correlates greatly with its absolute potential values. Negative Zeta-potential shows anion’s presence in the solution and higher absolute potential values come with more stable emulsions. According to Figure 2A, the Zeta-potentials of SPI/pectin were 17.94 ± 3.15 mV, −3.95 ± 1.19 mV, −28.07 ± 2.82 mV, −21.90 ± 0.82 mV and −26.68 ± 4.70 mV with pH at 3.0, 5.0, 7.0, 7.5, 9.0, respectively. With increasing pH value, the absolute potential underwent decrease and then increase. Negatively charged pectin molecules enclosed the SPI particles to form an external layer and the repulsion of charges (SPI/pectin complex particles) made the particles stable in the solution. At low pH condition, the particles were charged by protonation and the like positive charges repulsed another one to keep the stability. But when pH increased and closed to isoelectric point, the repulsion of charges was reduced sharply and therefore flocculation occurred. With pH increased continuously, electrostatic interaction between the charges was reinforced, repulsion shifted from positive charges at lower pH to negative charges at higher pH and the solution became stable again. However, things changed in olive oil-SPI/pectin emulsions. As Figure 2B illustrated, the Zeta-potentials of emulsion particles were −27.26 ± 1.60 mV, 4.04 ± 1.93 mV, −34.54 ± 2.86 mV, −37.08 ± 0.88 mV and −39.01 ± 2.65 mV with pH at 3.0, 5.0, 7.0, 7.5, 9.0. The absolute value of Zeta-potential firstly decreased and then increased with increasing pH value. Obviously, it was the turning point with minimum absolute potential value when pH was at 5.0 as the result of minimum particle charge attached when it closed to the electric point. Ionization equilibrium between amino and carboxyl affected the particle charge which directly impact on the emulsion’s stability. Coalescence occurred when repulsion interaction reduced within particles and then the emulsion lost stability. But being far away the electric point under other pH condition, it shared higher absolute potential value giving reinforced charge repulsion interaction and so the emulsion solution could maintain stable still. That is, keeping lower pH condition (pH 3.0) or higher pH condition (pH 9.0) would boost the stability of olive oil-SPI/pectin emulsion.

### 3.3. Microstructure and Particle Size Distribution of Olive oil-SPI/Pectin Emulsion

**Figure 3:**
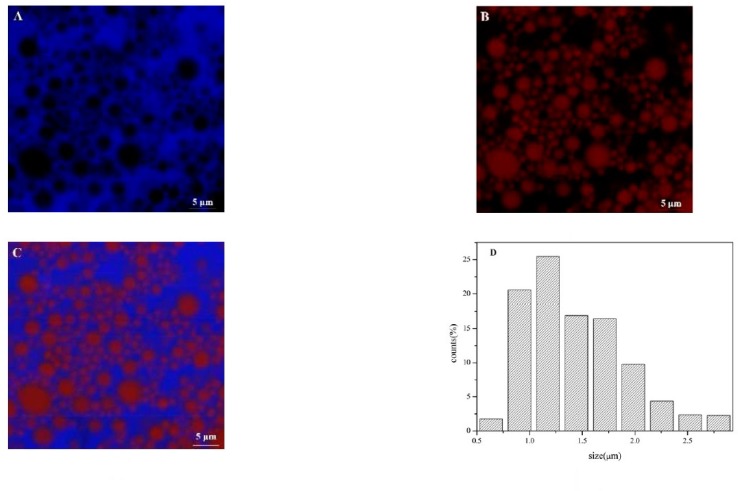
Confocal laser scanning microscopy (CLSM) diagram of the Pickering emulsion ((**A**): Nile Blue-stained soy protein isolate; (**B**): Nile red-stained olive oil; (**C**): Mixed-stained image) and potential solution of composite solution at different pH values; and (**D**) particle size dispersion of Pickering emulsion.

The microstructure of emulsion at pH3.0 was given in Figure 3. The confocal images showed protein particles completely absorbed on the oil-water interface. A thin and compact interface film formed whilst olive oil was entirely coated by the protein particles. Remaining protein helped shape the network structure in the continuous phase to prevent the collision and aggregation of oil particles that would cause aligned oil particle dispersion and stable emulsion. Uniform particle size was observed, and it changed from 0.5–3.5μm with mean particle size 1.42 ± 0.54 μm.

### 3.4. Rheological Behavior of Oil-SPI/Pectin Emulsion

**Figure 4:**
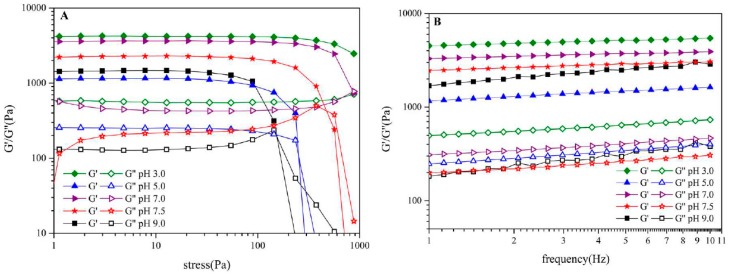
Rheology diagram of olive oil-SPI/pectin Pickering emulsion at different pH values ((**A**), stress scan; (**B**), frequency sweep).

Pickering emulsion often shares high rheological property which is dependent on its continuous phase. Droplets would squeeze with another one in high viscous emulsion leading to space in continuous phase narrowed down or even disappeared and then giving the emulsion higher viscoelasticity. Gel structure of emulsion generally correlated with its rheological property and hence, to determine the emulsion’s gel structure and stability, stress scan and frequency sweep were executed to illustrate the emulsion’s rheological behavior and to determine the emulsion’s linear rheological region, viscous modulus (G’’), elasticity modulus (G’). Viscous modulus and elasticity modulus intersect at critical stress (yield point) which refers to emulsion’s structure changed from gel to sol. According to Figure 4A, the emulsion at series of pH within linear rheological region represented gel property (G’ > G’’) and yield point occurred as stress increased. Emulsion at pH3.0, 7.0 and 7.5 had broad linear rheological zone and yield point had not occurred until stress was increased to 1000 Pa for emulsion at pH3.0, which suggested that emulsion at pH3.0 had strong elasticity property. Clearly, pH value was responsible for the emulsion’s gel property that made it possible to get improved get network by tuning the emulsion’s pH value. In addition, it has been reported that emulsion with excellent gel property shares elasticity modulus about 1000 Pa while yield point here occurred at stress 150 Pa within elasticity modulus region 1200~4200 Pa indicating excellent stability, elasticity and gel property which was likely accounted by using ultrasonic treatment to prepare samples. Frequency sweep diagram (Figure 4B) shows that elasticity modulus G’ were greater than viscous modulus G’’ from all emulsions. Frequency took greater responsibility for G’’ than G,’ suggesting the presence of solid-like rheological properties [42]. Elasticity modulus G’ increased at early stage and then went on decrease along with increasing pH value. Once more, it told that pH affected the gel property and lower pH value meant stronger gel property.

### 3.5. Storage Stability and Freeze-Thaw Stability of Olive Oil-SPI/Pectin Emulsion

**Figure 5:**
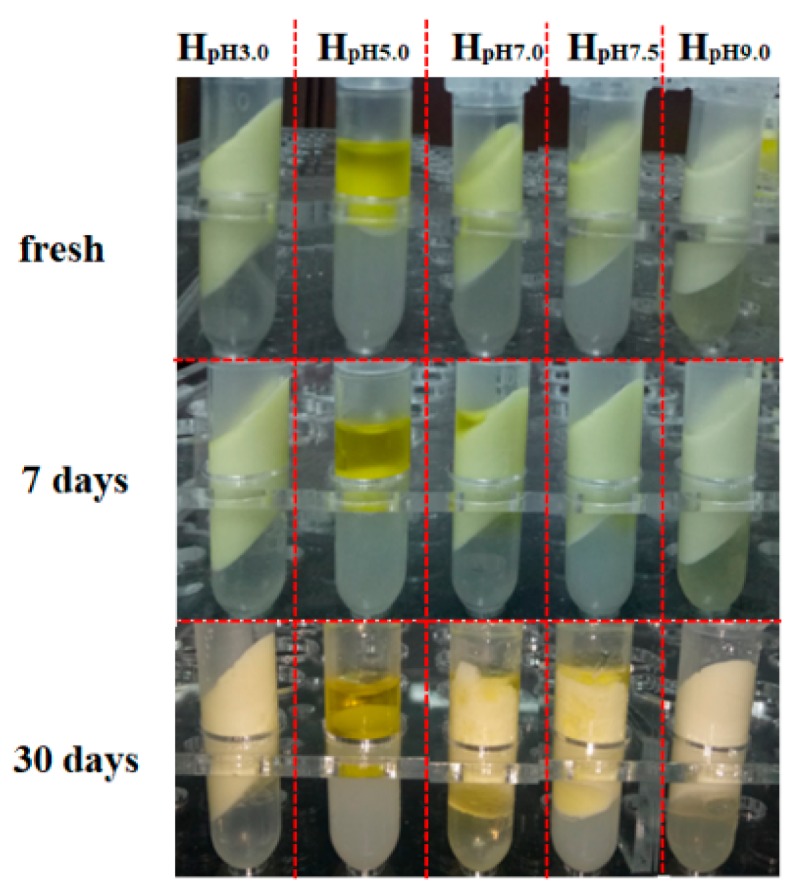
Apparent appearance of olive oil-SPI/pectin Pickering emulsions during storage process.

Firstly, the storage stability of emulsions at different pH was evaluated by apparent appearance upon storage of 30 days at 4 °C, as illustrated in Figure 5. Freshly prepared emulsions represented good stability except sample at pH5.0 underwent oiling off for lacking charge repulsion at its isoelectric point. After 7 days, oiling off of emulsion was intensified at pH 5.0 and occurred at pH 7.0, 7.5 as well. As showed by emulsion breaking, worsened in the order pH5.0 > pH7.0 > pH7.5 whilst other samples maintained stable. After 30 days, oiling off appeared in all samples except that at pH3.0. Apparently, emulsions at lower pH displayed better storage stability (sample at isoelectric point omitted) and got greatest gelation at pH 3.0. Soy protein isolate/pectin complexes have excellent emulsifying ability and at lower pH condition, it shows more favorable emulsification and interfacial absorption. Actually, different pH condition also means different interface tension which directly function at emulsion’s stability [43].

**Figure 6:**
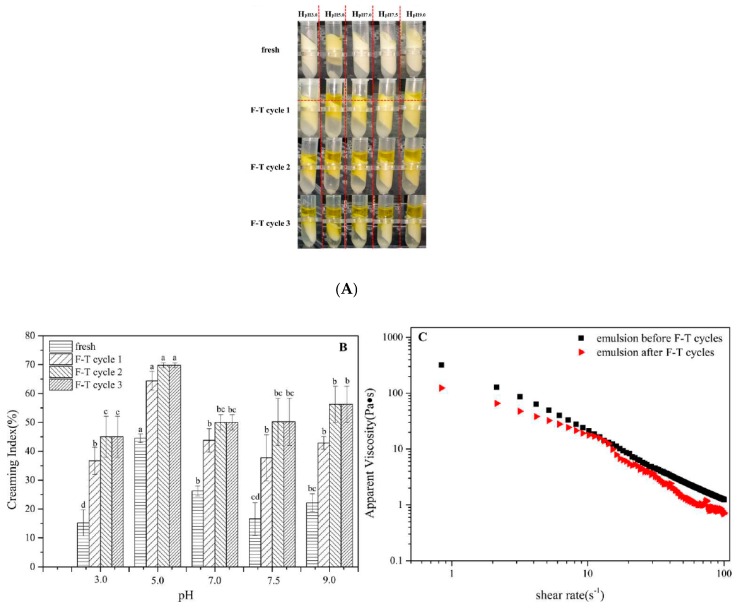
Olive oil-SPI/pectin Pickering emulsion treated with three freeze-thaw (F-T) cycles (**A**) apparent appearance; (**B**) creaming index of emulsions at pH series before and after freeze-thaw cycles. Data represent the mean ± one standard deviation (*n* = 3). Different lower-case letters represent a significant difference among pHs and freeze-thaw cycles; and (**C**) apparent viscosity of emulsion at pH3.0 before and after freeze-thaw cycles at 25 °C.

Freeze-thaw stability of emulsions was investigated subsequently. It was found that stratification extent of fresh emulsions increased with increasing pH value (except isoelectric point) which was due to flocculating improved at higher pH giving greater lipid-buoyance-resistance (Figure 6A). After one cycle, emulsion at pH3.0 represented the lowest creaming index~15%. Emulsion breaking occurred greatly at other pH conditions higher creaming index shown in Figure 6B. With more freeze-thaw (F-T) cycle treatment, stratification was reinforced because flocculating would be enhanced during repeated freeze-thaw cycles and droplets aggregated to form larger ones. In the way of gravity, it was more favorable for stratification to arise. Compared to samples at pH5.0~9.0, emulsion at pH3.0 displayed slightest stratification showing greatest freeze-thaw stability after three F-T cycles with ~45% creaming index. Moreover, as Figure 6B depicted, creaming index change decreased with increasing F-T cycles also indicating stratification stability was improved with F-T process. 

Higher viscosity means lower mobility ratio of droplets that can lead to collision and thus gives more resistance to instability caused by droplets aggregation. Shear viscosity is another factor for interfacial stability of emulsion. Newtonian fluid generally shares shear thinning, a pseudoplastic phenomenon, referring to the apparent viscosity decreases with increasing shear rates. According to Figure 6C, shear thinning occurred along with the increased shear rates in the sample at pH3.0 both before and after F-T cycles, which is contributed to flocculation structure in the emulsions. However, there was jump point phenomenon in the F-T cycles in that oiling off occurred during F-T cycle and free oil droplets enhanced the lubrication of emulsion particles, which reduced the emulsions viscosity. The results above revealed that olive oil-SPI/pectin emulsion at different pH were prepared and the emulsion system represented good gel structure, rheological behavior, storage stability and freeze-thaw stability, notably, the stability was pH-dependent.

### 3.6. FT-IR Spectrometer Analysis of Olive Oil-SPI/Pectin Emulsion with Embedded Quercetin

**Figure 7:**
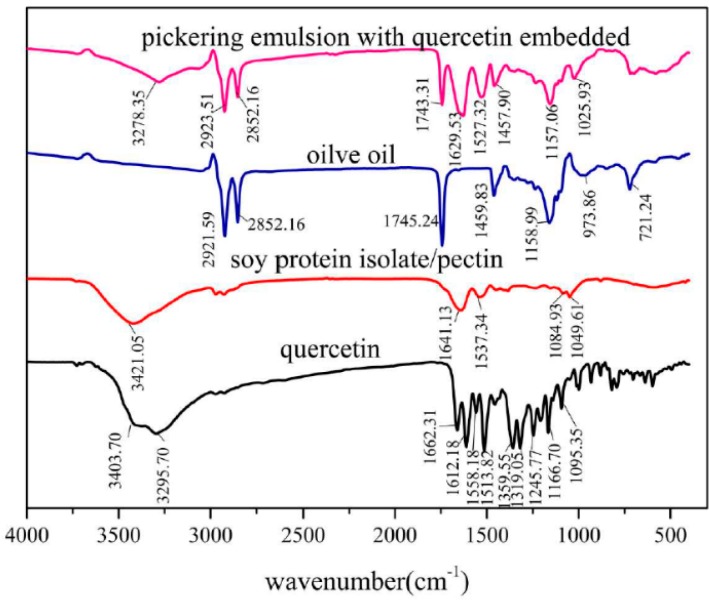
Fourier transform infrared (FTIR) spectra of pure quercetin, SPI/pectin complex particle, pure olive oil and quercetin embedded emulsion.

Infrared spectrum of quercetin embedded emulsion in comparison to individual component were depicted in Figure 7. It revealed that peak at 3404 cm^−1^ refers to stretching absorption from associated hydroxyl group in pure quercetin and 1612 cm^−1^ to C=O stretching, 1246 cm^−1^ to C-H stretching and 1167 cm^−1^ is derived from C-O-C stretching. For the olive oil-SPI/pectin emulsion containing quercetin, the N-H bending in amide II from soy protein isolate at 1537 cm^−1^ shifted to 1527 cm^−1^; peak at 1629 cm^−1^ referred to shifted C=O stretching in amide I from SPI at 1649 cm^−1^ accompanying shifted C=O stretching from quercetin at 1612 cm^−1^. Meanwhile, peak at 1743 cm^−1^ is contributed to olive oil (C=O stretching), 2923 cm^−1^ from both olive oil(C-H stretching at saturated skeleton) and 2852 cm^−1^ from olive oil (C-H stretching at saturated skeleton) as well. In particular, the peak at 3400~3420 cm^−1^ for phenolic hydroxyl group in quercetin was absent along with presence of new broad absorption band in 3000~3500cm^−1^ which is contributed to hydrogen-bond interaction between quercetin (-OH) and pectin (-COOH) demonstrated quercetin had been successfully embedded in the emulsion droplet rather than merely placed on the surface of emulsion particle.

### 3.7. Microstructure and Encapsulation Efficiency of Olive Oil-SPI/Pectin Emulsions with Embedded Quercetin

**Figure 8:**
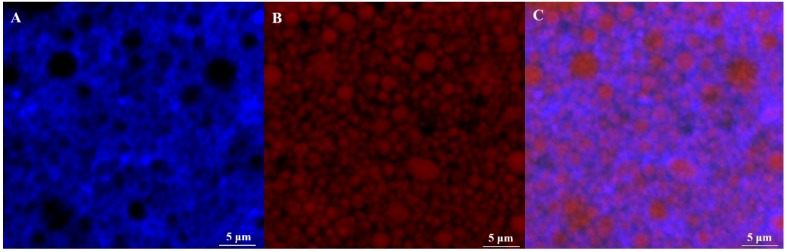
CLSM plot of emulsion with embedded quercetin ((**A**): Nile Blue stained soy protein isolate; (**B**): Nile red stained olive oil; (**C**): Mixed stained image).

**Figure 9:**
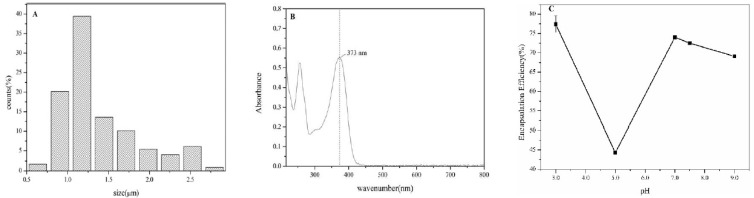
(**A**) Particle size distribution of emulsion with embedded quercetin; (**B**) maximum absorbance of quercetin in 300~800 nm; (**C**) pH-dependent encapsulation efficiency of quercetin in olive oil-SPI/pectin emulsion. Data represent the mean ± one standard deviation (*n* = 3). Different lower-case letters represent a significant difference among pHs.

CLSM observation of quercetin embedded emulsion system revealed that more regular distribution of particles had been obtained in comparison with emulsion containing no quercetin (Figure 8). This is due to quercetin improved the interaction between emulsion droplets and then promoted to form network structure in the system. The particle size remained unchanged and the average size was 1.43 ± 0.49 μm (Figure 9A). To measure encapsulation efficiency, the maximum absorbance was first determined and it was turned out to be at λ = 373 nm for quercetin, which was consistent with the literature [4]. Absorbance of quercetin was in directly proportion to its concentration, yielding to linear equation y = 0.0329x − 0.0011 (R^2^ = 0.9997). Encapsulation efficiency of quercetin was highly pH-dependent, as Figure 9C illustrated, it got highest encapsulation at pH3.0, 77.39% ± 2.15. Except for the unstable emulsion at isoelectric point (pH5.0), 44.29% ± 0.41, it was showed a tendency that emulsion containing quercetin at lower pH were more stable and also had higher encapsulation efficiency. The encapsulation efficiencies were 74.01% ± 0.32, 72.50% ± 0.40, 69.10% ± 0.15 at pH7.0, pH7.5 and pH9.0, respectively. Lower pH emulsions are more suitable for encampsucating quercetin.

### 3.8. Rheological Behavior of Olive Oil-SPI/Pectin Emulsion with Embedded Quercetin

**Figure 10:**
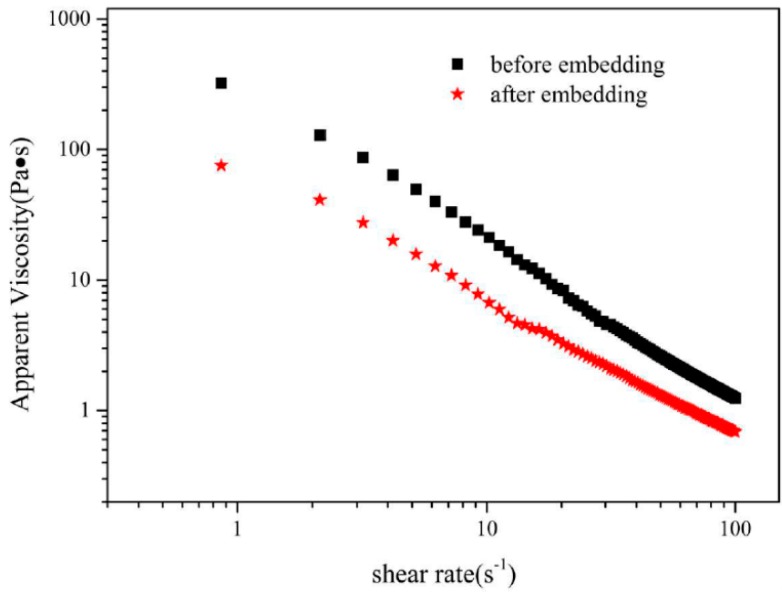
Apparent viscosity of emulsion before and after embedding quercetin (temperature: 25 °C).

Similar rheological behavior of emulsion containing quercetin was observed that shear thinning phenomenon occurred as shear rates increased. Whether quercetin was embedded or not, the emulsion system was Newtonian fluid (Figure 10). Notably, apparent viscosity of emulsion containing quercetin declined faster than that of no quercetin owning to interaction of hydroxyl group in quercetin with amino in protein influenced the complex particles’ surface property and then impacted on the emulsion’s viscosity to some extent. Figure 10 shows the apparent viscosity change of emulsion before and after embedding quercetin with a changing shear rate at 25 °C; it can be seen that the change will be related by equation $y_{1}=-1.0746\times x_{1}+2.2701\left(R^{2}=0.968\right)$, where $y_{1}$ is log Apparent Viscosity (Pa•S) and $x_{1}$ is log Shear Rate (S^−1^) for emulsion before embedding quercetin and also will be related by equation, $y_{2}=-0.9168\times x_{2}+1.6936\left(R^{2}=0.9749\right)$ where $y_{2}$ is log Apparent Viscosity (Pa•S) and $x_{2}$ is log Shear Rate (S^−1^) for emulsion after embedding quercetin.

### 3.9. Bioavailability Analysis of Quercetin Stabilized by Olive Oil-SPI/Pectin Emulsion 

**Figure 11:**
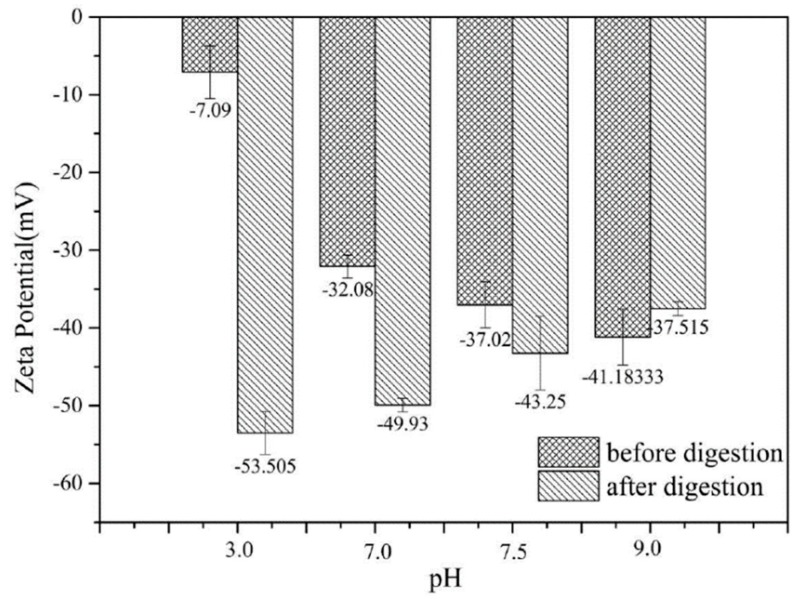
Zeta-potential of emulsion with embedded quercetin before and after in vitro digestion. Data represent the mean ± one standard deviation (*n* = 3).

In order to evaluate the bioavailability of quercetin stabilized by olive oil-SPI/pectin emulsion at series of pH, simulated in vitro gastrointestinal digestion was conducted (with pure olive oil as counterpart). Juice was extracted for determination when intestinal digestion stage was completed. According to the zeta-potential diagram before and after digestion (Figure 11), absolute potential value increased in samples at pH3.0–9.0 (with isoelectric point omitted) after digestion. At pH 3.0, the absolute potential was abruptly increased from 7.09 mV to 53.51 mV after digestion, while moderate change was observed at pH7.0 and pH7.5, from 32.08 mV to 49.93 mV and 37.02 mV to 43.25 mV, respectively. Lipase hydrolyzed olive oil to give free aliphatic acid and glycerol stearate with negative charge resulting net charge added in the digestive juice. Meanwhile, soy isolate protein would be replaced with negative charged cholate, lipid particles absorbed on the oil-water interface causing more anions, much fewer local anions like cholate, OH^−^, Cl^−^ in the digestive juice would combine to the emulsion particle, which all function synchronously to make more charge detected.

It is a simple but efficient measure to determine the lipolysis extent of lipid during digestion test by calculating the release rate of free fatty acids. Release rate of free fatty acids during digestion process was monitored by NaOH consumption, which was summarized in Table 1. When intestinal digestion stage finished, the amount of NaOH consumption showed a tendency, that is, pure oil > HQ_pH3.0_ > HQ_pH7.0_ > HQ_pH7.5_ > HQ_pH9.0_. In digestion process lipid broke up to oil-water solution by physical collision or surfactant function (like cholate, phospholipid) and subsequently underwent lipolysis by lipase that absorbed on the oil-water interface. Therefore, the superficial environment of oil droplets and its phy-chemical property really play an essential role in the lipolysis. In pure oil, there was large interfacial area enabling more lipase to be absorbed on the surface and hence, greater lipolysis was observed in pure olive oil, up to 29.40%. However, in emulsion system lipase need to across oil-water interface and diffuse in the oil phase in succession to degrade the lipid, hence, stable emulsion system can efficiently inhibit the degrade rate of lipid. Emulsion at different pH shared different stability and oiling off, coalescence would speed up the diffusion of lipase and give higher lipolysis extent. Lipid hydrolysis in digestion causing embedded quercetin released that makes quercetin on this stage be able to be used. Rate and extent of lipid hydrolysis are factors for bioavailability. Absorbance of emulsion juice after digestion was investigated to measure bioavailability at different pHs. As depicted in Table 2, availability for samples at pH7.0 was the greatest, 15.49% and the second one is 7.8% at pH3.0. It decreased in order, that HQ_pH7.0_ > HQ_pH3.0_ > HQ_pH7.5_ > HQ_pH9.0_ > pure oil. The results revealed that the pH need to be adjusted at 3.0 to maintain the emulsion stable for a long period and to take better advantage of quercetin, the pH7.0 is preferred.

## 4. Conclusions

This work demonstrated the preparation of olive oil-soy protein isolate/pectin emulsion with quercetin embedded by ultrasound treatment. It was found that within detected pH range 3.0–9.0, lower pH means more stable emulsion system. Emulsion at pH 3.0 remained stable after 30 days’ storage at 4 °C and exhibited best freeze-thaw stability after 3 cycles. Rheological behavior examination revealed emulsion at pH3.0 had broad viscoelasticity zone and has the best viscoelasticity stability as well. Those olive-oil isolate/pectin emulsions encapsulated quercetin with high encapsulation efficiency, up to 77.39%. In vitro intestinal digestion was conducted, and quercetin availability reached 15.94% at pH 7.0 and 7.8% at pH 3.0. This research provided a solution to prepare olive oil-soy protein isolate/pectin emulsion and correlated its stability with pH condition. The olive oil-SPI/pectin emulsion exhibited good quercetin availability applying for an option to take advantage of quercetin in a green and salutary way.

## Figures and Tables

**Table 1:** Release rate of free fatty acids (NaOH consumption) of olive oil-SPI/pectin emulsion with embedded quercetin during in vitro gastrointestinal digestion.

Simulated Digestion	In Vitro Gastrointestinal Digestion				
Samples	Pure Olive Oil	HQ_pH3.0_	HQ_pH7.0_	HQ_pH7.5_	HQ_pH9.0_
Release rate (%) of free fatty acids	29.40	16.35	15.40	13.50	11.75

**Table 2:** Bioavailability of quercetin stabilized by emulsion at different pH.

	Before Digestion					After Digestion				
Samples	Pure Oil	HQ_pH3.0_	HQ_pH7.0_	HQ_pH7.5_	HQ_pH9.0_	Pure Oil	HQ_pH3.0_	HQ_pH7.0_	HQ_pH7.5_	HQ_pH9.0_
Retention rate of quercetin%	100.00	77.39	74.01	72.50	69.10	98.25	69.59	58.52	66.80	66.33
Bioavailability%	-	-	-	-	-	1.75	7.80	15.49	5.70	2.77
